# Adaptive evolution of West Nile virus facilitated increased transmissibility and prevalence in New York State

**DOI:** 10.1080/22221751.2022.2056521

**Published:** 2022-03-31

**Authors:** Sean M. Bialosuknia, Alan P. Dupuis II, Steven D. Zink, Cheri A. Koetzner, Joseph G. Maffei, Jennifer C. Owen, Hannah Landwerlen, Laura D. Kramer, Alexander T. Ciota

**Affiliations:** a New York State Department of Health, The Arbovirus Laboratory, Wadsworth Center, Slingerlands, NY, USA; b Department of Biology, State University of New York at Albany, Albany, NY, USA; c Department of Fisheries and Wildlife, Michigan State University, East Lansing, MI, USA; d Department of Biomedical Sciences, State University of New York at Albany School of Public Health, Albany, NY, USA

**Keywords:** West Nile virus, virus evolution, arbovirus, Flavivirus, adaptive evolution

## Abstract

West Nile virus (WNV; Flavivirus, Flaviviridae) was introduced to New York State (NYS) in 1999 and rapidly expanded its range through the continental United States (US). Apart from the displacement of the introductory NY99 genotype with the WN02 genotype, there has been little evidence of adaptive evolution of WNV in the US. WNV NY10, characterized by shared amino acid substitutions R1331K and I2513M, emerged in 2010 coincident with increased WNV cases in humans and prevalence in mosquitoes. Previous studies demonstrated an increase in frequency of NY10 strains in NYS and evidence of positive selection. Here, we present updated surveillance and sequencing data for WNV in NYS and investigate if NY10 genotype strains are associated with phenotypic change consistent with an adaptive advantage. Results confirm a significant increase in prevalence in mosquitoes though 2018, and updated sequencing demonstrates a continued dominance of NY10. We evaluated NY10 strains in *Culex pipiens* mosquitoes to assess vector competence and found that the NY10 genotype is associated with both increased infectivity and transmissibility. Experimental infection of American robins (*Turdus migratorius*) was additionally completed to assess viremia kinetics of NY10 relative to WN02. Modelling the increased infectivity and transmissibility of the NY10 strains together with strain-specific viremia demonstrates a mechanistic basis for selection that has likely contributed to the increased prevalence of WNV in NYS.

## Introduction

West Nile virus (WNV; *Flavivirus*, *Flavivridae*) is a mosquito-borne single-stranded, positive sense RNA virus with a genome of approximately 11 kb encoding a single open reading frame (ORF) consisting of three structural genes (*C*, *prM*, and *E*) and seven non-structural genes (*NS1*, *NS2A*, *NS2B*, *NS3*, *NS4A*, *NS4B*, and *NS5*) [1]. WNV was first isolated from a febrile viremic patient in Uganda in 1937 and subsequently caused isolated outbreaks in Africa, the Middle East, and Australia, where the disease was rarely found to be neuroinvasive [2]. In the mid-1990s the intensity of outbreaks and WNV disease increased, marked by rising prevalence in Eastern Europe and Northern Africa [3]. WNV is now the most geographically widespread arbovirus and has been classified into as many as five genetically disparate lineages that differ by as much as 20-25% nucleotide identity [4]. The introduction of lineage 1 WNV to the United States (US) commenced in New York State (NYS) in 1999 [5]. There have been over 55,000 human cases diagnosed in the US since 1999, including over 2600 deaths [6]. Although most cases of WNV are subclinical, roughly 20% of cases progress to acute febrile illness, and 1% of cases progress to central nervous system (CNS) infection [7]. CNS infection results in a far more severe course of disease, marked by a range of clinical outcomes including encephalitis, meningitis, acute flaccid paralysis and death [8]. Given both the high proportion of subclinical infections and the fact that West Nile fever cases often go undiagnosed, the true number of infections in the US has likely exceeded 6 million over the last twenty years [9].

The exploitation of a naïve and permissive host environment together with highly competent vectors in North America facilitated rapid spread and establishment of WNV as the most prevalent arboviral pathogen in the US. WNV is maintained in an enzootic cycle between mosquitoes, primarily of the *Culex* genus, and avian hosts. The primary vector in Northeast US is *Culex pipiens*. Most passerine songbirds serve as reservoir hosts that amplify the virus to viremia levels sufficient for transmission back to the mosquito vector [10]. Despite the wide host breadth of WNV, American robins (*Turdus migratorius*) are known to play a disproportionally large role in amplification and dispersal, both because of their competence and the blood-feeding preferences of *Culex spp*. mosquitoes, as well as their migratory habits and short distance movements [11–14].

As an RNA virus with no proofreading mechanisms and a high rate of replication, WNV has enormous evolutionary potential, yet estimates of evolutionary rate ranging from 3.6×10^−4^–8.2×10^−3^ substitutions/site/year stand in contrast to this lack of fidelity in WNV genome replication [15–17]. While WNV has been relatively stable genetically, high levels of variability with largely uncharacterized phenotypic consequences have been noted over various temporal and geographic scales using geographically focal datasets [18–20]. In addition, evidence of adaptive evolution of WNV is scant, with notable exceptions. The invasive WNV strain, introduced to the US in 1999, possessed a characteristic amino acid substitution, NS3 T249P, which increased virulence and susceptibility in avian hosts [21]. Displacement of the previous NY99 genotype by the WN02 genotype, characterized by a single amino acid change in the envelope protein, V449A, likely contributed to the rapid dispersal of the virus across the US [22]. The WN02 genotype was found to be more infectious to mosquitoes, demonstrating earlier dissemination and a shorter extrinsic incubation period (EIP) in *Culex tarsalis*, which is widespread in the US west of the Ohio River [23]. An additional genotype, SW/WN03, characterized by the amino acid substitutions NS5 K314R and NS4A A85T, has been circulating in the US since 2003 [24].

Recent phylogenetic studies of WNV identified multiple mutations with evidence of positive selection and novel genotypes that have increased in prevalence in recent years in NYS [25]. In particular, the NY10 genotype, characterized by two shared amino acid substitutions with evidence of positive selection, R1331 K (NS2A R188 K) and I2513M (NS4B I240M), emerged in NYS in 2010 and increased in prevalence through 2015. Importantly, this displacement occurred in concert with increased WNV activity in the state, a trend that continued through 2018. Here, we sequenced an additional 48 WNV strains isolated from 2015–2018 to confirm the continued dominance of NY10. To test the hypothesis that adaptive evolution contributed to increased WNV transmission and prevalence, we characterized NY10 strains *in vivo* in both *Cx. pipiens* and American robins. Using these results, we modelled WNV transmissibility and demonstrated a clear role for viral genotype in driving WNV activity in the region.

## Methods

### West Nile virus mosquito surveillance and sample preparation

Mosquitoes were collected in Centers for Disease Control (CDC) light traps by NYS county health departments and speciated pools were submitted to the NYS Arbovirus Laboratory for processing and testing. Pools consisted of 15 - 60 *Cx. pipiens* and/or *Cx. restuans* females in 1 mL mosquito diluent [MD, 20% heat-inactivated fetal bovine serum (FBS) in Dulbecco’s phosphate-buffered saline (PBS) plus 50 μg/mL penicillin/streptomycin, 50 μg/mL gentamicin, and 2.5 μg/mL Fungizone] with 1 steel bead (Daisy Outdoor Products, Rogers, AR). Pools were processed by homogenization for 30 s at 24 Hz in a Mixer Mill MM301 (Retsch, Newtown, PA), followed by centrifugation at 6000 rcf for 5 min. WNV-positive pools were identified by quantitative real-time reverse transcription polymerase chain reaction (qRT-PCR) [26]. WNV prevalence was determined using maximum likelihood estimation (MLE) based on mosquito surveillance pool sizes using an Excel Add-In (https://www.cdc.gov/westnile/resourcepages/mosqSurvSoft.html). Geographically and temporally representative pools (Table 1) were amplified on Vero cell culture (African green monkey, *Chlorocebus sabaeus*, ATCC, Manassas, VA) and the resulting supernatant was saved for subsequent characterization [27]. RNA was extracted on the MagMax-96 Express robot (Applied Biosystems, Foster City, CA) with the Magmax Viral isolation kit (ThermoFisher Scientific, Waltham, MA), according to manufacturer’s recommendations with modifications. Briefly, 50 μL of supernatant samples were added to 130 μL of lysis buffer containing 20 μL of RNA binding beads that were diluted 1:1 with wash buffer 1. RNA was eluted in 90 μL of elution buffer. Primer pairs, AGTAGTTCGCCTGTGTGAGCTGAC, GAGAGCCCCCAGCAATCC, and CCTTGCAAAGTTCCTATCTC, CTCTGCCAGCCCTCCGACGAT, and GGACCAACCAGGAGAACATTT, GATCCGAGTACACCCTGGCGTCAA, and CAAGGCGAGCAGGGTGAT, GAAGCTCGACTCACCCAATACAT, and GCTCTGCCCCTACATGCCGAAAGT, CGGCTGAGTCTTTCTTCCCCATTC, and TGAGGAGCGCGAGGCACAT, AGATCCTGTGTTCTCGCACCACCAGC were used to generate 6 overlapping fragments of approximately 2.5 kb each using One-step superscript III RT–PCR with platinum TAq (Life Technologies, Carlsbad, CA). Six separate reactions were performed using 5 μL of RNA, 1 μL of enzyme, and 0.4 μM final concentration of primer pairs in a total reaction volume of 25 μL. Products were amplified using a thermocycler and the following conditions: 55 °C for 30 min; 94 °C for 2 min; 40 cycles of 94 °C for 15 sec, 55 °C for 30 sec, 68 °C for 3.5 min; and a final extension of 68 °C for 10 min (Simpliamp by Applied Biosystems, Waltham, MA). Two uL of amplicons were visualized on a 1% agarose gel, after which the same amplicons of RT–PCR reactions for each sample were pooled and purified using Zymo DNA Clean and Concentrate (Zymo Research, Irvine, CA). Amplicons from individual isolates were pooled and sent to Wadsworth Center Applied Genomics Technology Core (WCAGTC) for library preparation and indexing using the Nextera XT kit (Illumina, San Diego, CA) according to manufacturer's protocols.

**Table 1.** Table of newly sequenced strains isolated by county and year (*n* = 48).

	2013	2014	2015	2016	2017	2018	Total
Cattaraugus	0	0	0	0	0	2	2
Clinton	0	0	0	0	0	3	3
Erie	2	0	2	2	1	1	8
Nassau	2	1	2	1	2	1	9
Onondaga	1	1	0	0	2	1	5
Orange	0	0	1	2	1	2	6
Rockland	1	2	0	1	1	0	5
Suffolk	0	0	1	1	0	2	4
Westchester	1	0	1	0	1	3	6
TOTAL	7	4	7	7	8	15	48

### Sequencing and genetic analyses

Sequencing was performed on the Illumina MiSeq platform (San Diego, CA). Paired-end reads were assembled to a WN02 genotype reference (DQ164190) deploying Geneious Pro’s reference mapping tool using high sensitivity and free end gaps with 10 iterations of fine tuning, trimming paired read overhangs. The same parameters were used to map reads to the consensus assembly. The newly sequenced strains were submitted to GenBank and assigned the accession numbers MT967988 – MT968032 and OK631659 – OK631661. All alignments were performed using MAFFT alignment in Geneious Pro, with the algorithm set to the slow and accurate L-INS-I alignment algorithm, with the scoring matrix set to 200PAM/K = 2. The gap open penalty was set to 1.53, and the offset value set to 0.123. Phylogenetic analyses were carried out using BEAST2 and all available NYS WNV sequences containing a full open reading frame (ORF) and assigned collection dates using available metadata (n = 590). Evolutionary rates were estimated using the Bayesian Markov chain Monte Carlo method implemented in the programme BEAST2 [28]. The GTR + I substitution model was found to be the best-fit for this dataset using bModelTest and all subsequent Bayesian analyses used these parameters [29]. A Gamma site model was assigned to the dataset, and a general time reversible (GTR) model was used to estimate substitution rates. A relaxed lognormal clock was used to estimate the evolutionary rate. A coalescent Bayesian skyline model was applied to the dataset and run for 800,000,000 generations, sampling every generation and discarding the first 10% of generations as burn-in. This number of generations was sufficient to ensure convergence and estimated sampling size (ESS) of all parameters of >200.

### Viruses

The WN02 strain used was isolated in 2003 (DQ164189) from an American crow (*Corvus brachyrhynchos*) found in Albany County, which was initially amplified on Vero cells for sequencing, and then later amplified on C6/36 cells (*Aedes albopictus*, ATCC, Manassas, VA) for downstream use.

Distinct NY10 genotype isolates were amplified on C6/36 cells to generate virus stocks for characterization. The NY10A (KX547330) and NY10B (KX547391) strains used were isolated from *Culex* mosquito surveillance pools from Erie County in 2013 and 2010, respectively. NY10C (KX547356), was isolated from a *Culiseta melanura* pool from Oswego County in 2012. Each strain possesses the signature, shared NY10 mutations in addition to unique nonsynonymous mutations (Table 2). After 5 days of amplification on C6/36 tissue culture, following an infection at ∼1.0 multiplicity of infection (MOI), culture supernatant was harvested and stored in 20% FBS at −80°C.

**Table 2.** Polyprotein position and unique amino acid substitutions in each of the West Nile virus strains utilized for experimental infections. The NY01 mutation is denoted here with an asterisk.

Position	Gene	NY99	WN02	NY10A	NY10B	NY10C*
178	prM	V		A		
499	E	V	A	A	A	A
584	E	K				R
1331	R			K	K	K
1400	NS2B	I			V	
1433	NS2B	D				
1991	NS3	F	L			
2377	NS4B	G				E*
2513	NS4B	I		M	M	M
2826	NS5	E		A		
3059	NS5	K	R			
3321	NS5	V				I

### Vector competence assays and infectivity studies

To assess WNV strain infectivity for the NY10 strains NY10A, NY10B, NY10C and WN02 we used *Cx. pipiens*, originally colonized from egg rafts collected in Pennsylvania in 2004 and subsequently maintained at the NYS Arbovirus Laboratory Insectary. Four-to-seven-day-old adult females were collected and fed on doses of WNV ranging from 5 to 8 log_10_ pfu/mL. Bloodmeals consisted of a 1:1 mixture of diluted virus stock and chicken blood (Colorado Serum Company, Denver, CO), and a final concentration of 2.5% sucrose. Following one hour of feeding using an artificial feeding chamber (Hemotek, Blackburn, UK) at 37 °C, mosquitoes were anesthetized, and the engorged females were collected and held at 27 °C for 11 days post-infection (DPI). Individual mosquitoes were saved with a 4.5 mm zinc-plated steel ball (BB) (Daisy, Dallas, TX) in 1.0 mL MD at −80°C. To determine infectivity, thawed samples were homogenized at 24 Hz for 30 s and subsequently tested by WNV-specific qRT-PCR [30]. A total of 50 mosquitoes were tested for each strain and dose combination. Infectivity curves were generated by plotting proportion infected and dose, and fitting log-linear curves using Graphpad Prism 9. Doses at which 50% of mosquitoes are infected (ID50s) were determined by extrapolating from these curves. Slopes were compared using linear regression analyses and proportions infected at individual doses were compared using ANCOVA tests via Graphpad Prism 9.

For vector competence assays, all WNV strains were diluted to 7.3 log_10_ pfu/mL in chicken blood and engorged females were held for 5 or 11 DPI and assayed for infection, dissemination, and transmission [23]. Legs were removed and stored at −80°C with a BB and 500 μL MD to assess dissemination. Transmission was determined by collecting saliva from anesthetized mosquitoes using *in vitro* transmission assays. Following 30 min of forced salivation, transmission fluid (1:1 FBS: 50% sucrose) was ejected into 150 μL MD and stored at −80 °C. All samples were tested by plaque screening on Vero cells and proportions of infected, disseminated, and transmitting were compared using Fisher’s exact tests using Graphpad Prism 9.

### Avian inoculations and viremia kinetics

All procedures and methods were approved by the Wadsworth Center Institutional Animal Care and Use Committee and trapping was completed and approved by Federal and State Scientific Collection Permits (SC1386, MB194270), and Master Banding Permit (#23269). Twenty-two hatch-year (HY) American robins were captured during fall migration from 12 - 22 October 2018 using mist nets (36 mm mesh; 12 m x 2.6 m) in Laingsburg, MI (42.82 - 84.38). Upon capture, the condition of each bird was assessed for body mass (± 0.1 g), sex, wing length, and presence of ectoparasites. Initially, birds were placed in individual wire cages (30 × 38 × 38 cm) until they acclimated, at which point they were moved to small aviaries (183 × 61 × 274 cm) with 2–4 robins in each. On 25 October 2018, robins were placed into bird holding boxes and transported via car from East Lansing, MI to Albany, NY. They were housed in one ABSL3 room at the NYS Arbovirus Laboratory in individual cages as described above. Room temperature was maintained at an average of 20–21 °C with 60% relative humidity and a 13-hour light: 11-hour dark photoperiod. All birds were fed a mixed diet appropriate for the species. Birds were provided *ad libitum* access to water throughout the entire experimental period. Prior to group assignment, all robins were screened for previous exposure to WNV by plaque reduction neutralization test (PRNT) upon capture and prior to experiments (∼14 days post capture). The blood samples were stored at 4°C until antibody titres were assayed. For the PRNT testing, sera were diluted in BA-1 [M199 medium with Hank’s salts, 1% bovine albumin, TRIS base (tris [hydroxymethyl] aminomethane), sodium bicarbonate, 2% FBS, and antibiotics] and heat-inactivated at 56 °C for 30 min. Sera were screened at a 1:10 dilution for WNV. Antibody titre was expressed as the inverse dilution of blood that neutralized 90% of the virus inoculum as compared to the virus-only control (no antibody) well [31]. Birds were randomly assigned to either WN02 (n = 10) or NY10 (n = 12) exposure groups and were subsequently inoculated subcutaneously in the cervical region with 0.1 mL of 5 log_10_ pfu/mL of infectious WNV (WN02 1986, NY10A, or NY10C), diluted in a sterile PBS diluent (PBS with 1% FBS). To assess viral titres, 0.05 mL blood was collected daily through 6 DPI, from the ulnar vein using a 25-gauge needle [31]. Blood was dispensed in BA-1 and stored at −80 °C. Viremia levels were subsequently quantified using the Vero cell plaque assay and compared among groups at each timepoint using t-tests [32]. At 14 DPI, all WNV-infected birds (control birds were held for a subsequent experiment not described here) were euthanized via CO_2_ asphyxiation.

### Infectivity and transmissibility indices

To assess the relative differences in the capacity for maintenance and spread of WN02 and NY10 strains, indices of infectivity and transmissibility were calculated. Avian infectivity (i_a_) was quantified using the viremia values for WN02 and NY10 strains (Figure 2B). Specifically, to account for the magnitude and duration of viremia, the area under the curve for each individual bird and strain was quantified for viremia levels of 4-5, 5-6, 6-7, 7–8 and >8 log_10_ pfu/mL, and mean values for WN02 and NY10 strains were obtained. Mosquito infectiousness (i_m_), which was quantified from mosquito infectivity experiments, was determined by extrapolation of mean levels of infection at the same blood meal titres from the linear relationship between dose and infection rates for each strain (Figure 4B). The product of i_m_ and i_a_ is infectiousness (i), at a given titre. Infectivity index, I, (Figure 7A) is defined as the sum of all i terms. Using transmission data from the vector competence experiments (Figure 6) a mosquito transmission term is introduced, t_m_, which is the proportion of infected mosquitoes transmitting either WN02 or NY10. The product of I and t_m_ equate to transmissibility, t, at a given titre, and the sum of all t terms equates to the overall transmissibility index, T (Figure 7B). This assessment of the infectivity and transmissibility indices allows us to estimate the relative capacity for emergent genotypes to displace other genotypes in the transmission cycle of WNV.

## Results

### West Nile virus surveillance and sequencing

**Figure 1.**
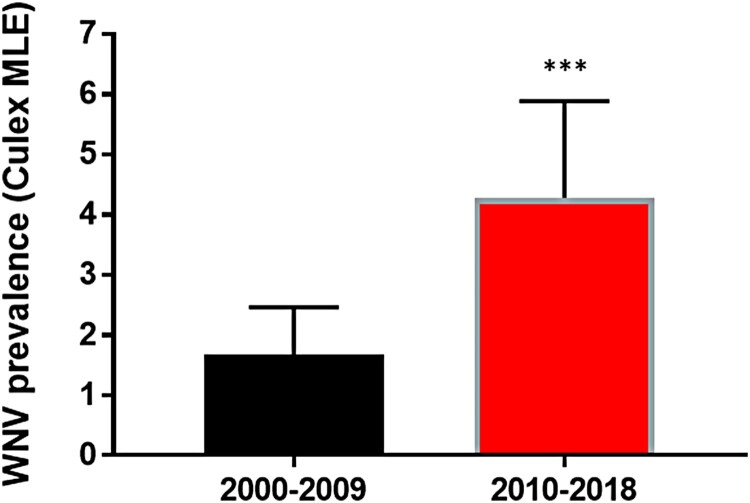
West Nile virus (WNV) prevalence in Culex mosquitoes in New York State (NYS). Prevalence (WNV positive/1000 tested) was calculated by maximum likelihood estimate (MLE) following pooling and testing of Culex mosquitoes by qRT-PCR. WNV prevalence was significantly greater from 2010–2018 relative to 2000–2009 (chi-squared test, ***p < 0.0001).

**Figure 2.**
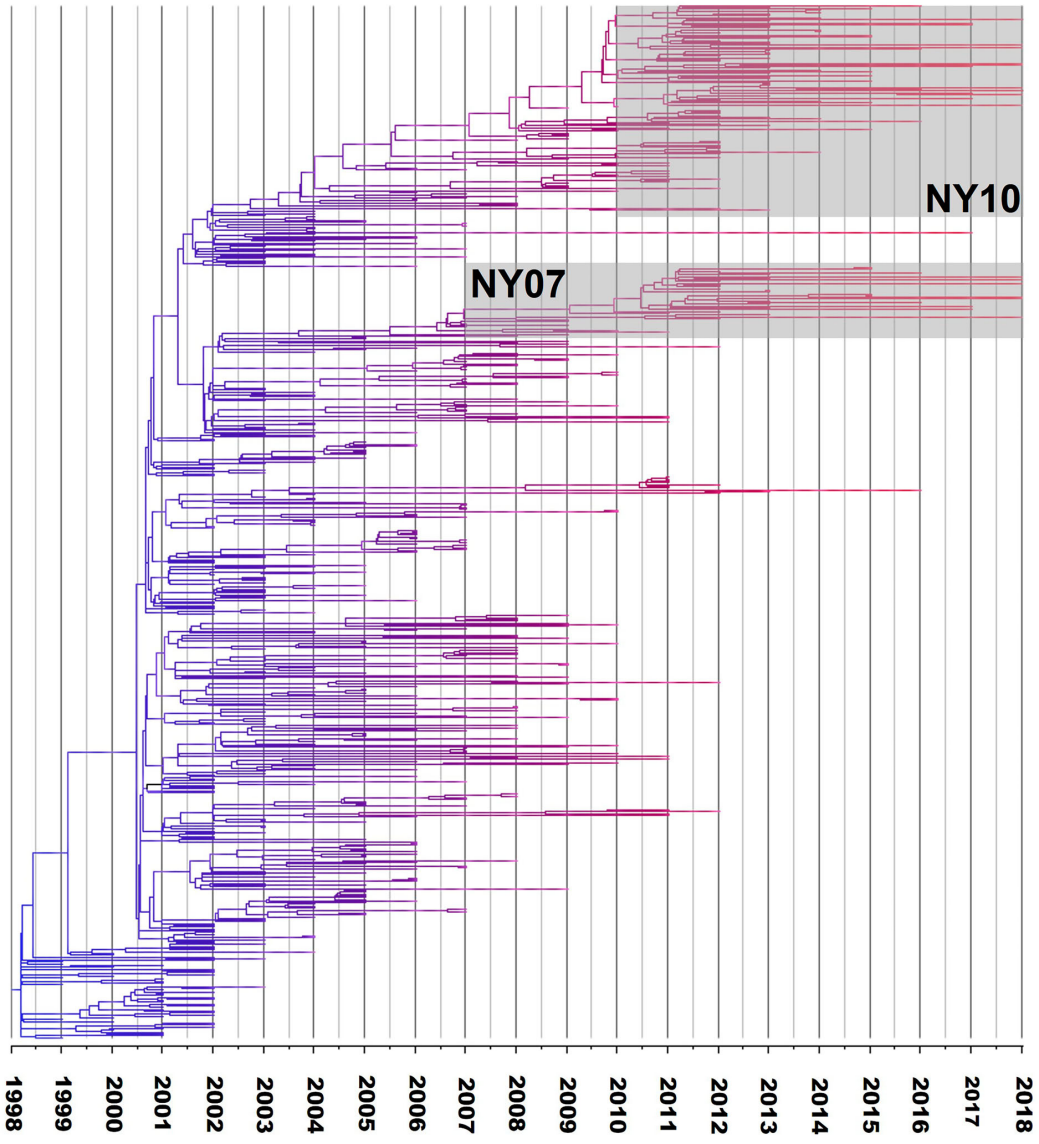
Time tree based on Bayesian analysis of West Nile virus (WNV) isolates from New York State (NYS), ranging from 1999–2018 (BEAST 2). Branch colours reflect the age of taxa, red branches represent the most recent strains. NY10 and NY07 genotype strains are enclosed in the respective boxes on the phylogeny.

**Figure 3.**
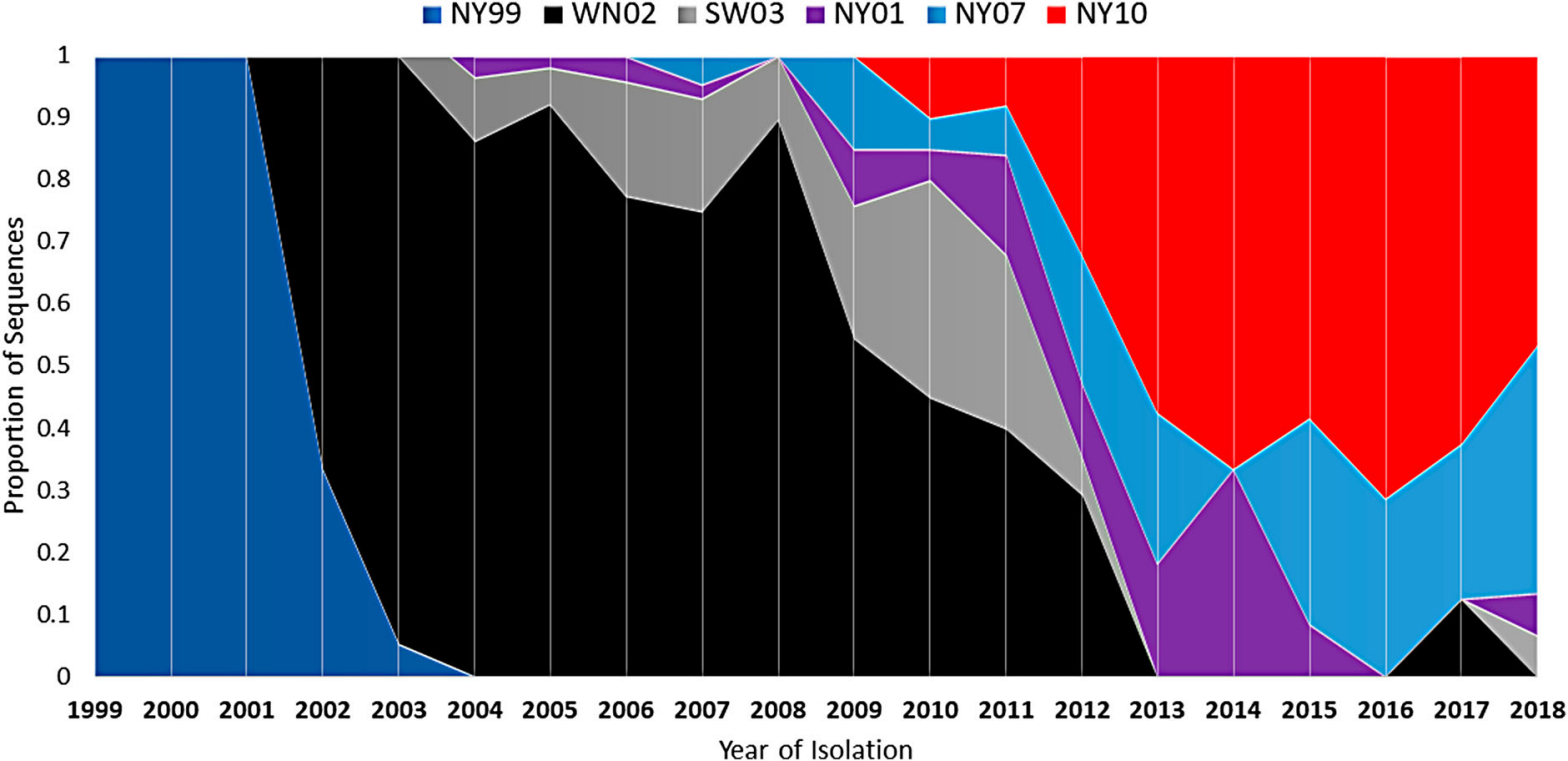
West Nile virus (WNV) genotype displacement in New York State (NYS), 1999-2018. Proportions of sequenced isolates belonging to distinct genotypes are shown. The displacement of the NY99 genotype by WN02 occurred from 2002-2004. NY01, NY07 and NY10 genotypes share a common WN02 backbone.

The NYS Arbovirus Laboratory tested a total of 70,547 *Cx. pipiens*-*Cx. restuans* pools from 2000 to 2009 and 58,615 pools from 2010 to 2018. A significantly higher positivity rate was measured from 2010–2018 (3,804/58,615, 6.5%) relative to 2000–2009 (1,769/70,547, 2.5%; Chi-squared, *p* < 0.0001). Estimates of prevalence based on pool size and positivity rates (MLE) were 1.67/1000 for 2000–2009 and 4.28/1000 for 2010–2018 (Figure 1). Updated sequencing efforts allowed for expansion upon previously observed trends in the genetic record established through mosquito surveillance in NYS. An initial displacement of the NY99 genotype, and fixation of the WN02 genotype, established a permanent change in the genetic record of circulating WNV genomes in the US, and the only selective sweep documented in North American strains of WNV. The characteristic WN02 mutation (E V449A) is present in all strains sequenced after 2003 and is the established genetic “backbone” of circulating WNV strains in the Americas. Of the 48 newly sequenced isolates, 33 were found to have the shared NY10 genotype amino acid substitutions (K1331R and I2513M) and 13 were found to have the shared NY07 genotype amino acid substitutions (T1195I, L1238F, S1838T, and S2287I). The NY10 genotype appears in 2010 and persists through 2018 (Figure 2). Three years after the emergence of NY10 it became the dominant genotype, a trend that has continued through 2018 (Figure 3). The prevalence of NY07 genotype strains has been more variable, yet there was an increase from 2016 to 2018. Other strains that were previously recognized as either of increasing prevalence in past years, or as showing evidence of mutations under positive selection, such as NY01 and SW/WN03, appear more ephemeral in their frequency yet persist through 2018 (Figure 3).

### Increased infectivity of West Nile virus NY10 genotype strains in Cx. pipiens

**Figure 4.**
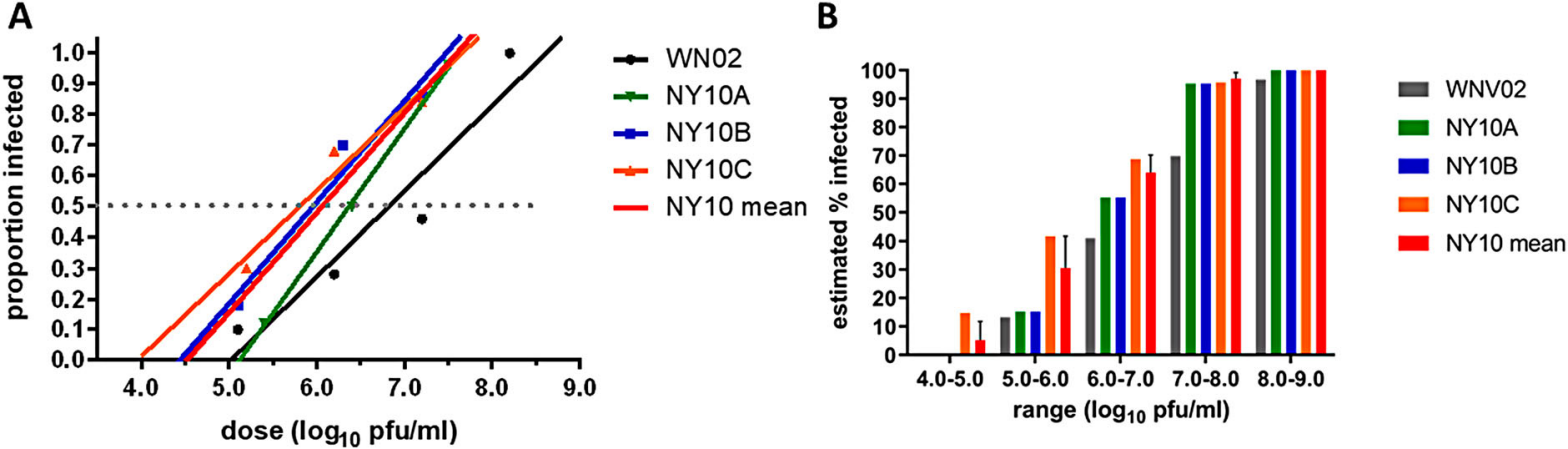
Mosquito infectivity of WN02 and NY10 strains in *Culex pipiens*. A) Proportion of mosquitoes infected at a given dose of WNV WN02 and NY10 strains. The titres at which 50% of mosquitoes are infected (ID50) are extrapolated and indicated by the dotted line. B) The estimated percent of infected *Culex pipiens* based on experimental results at each indicated range of input titres.

When considering the dose-dependent effects of individual strains on mosquito infection, a clear trend emerged in the proportion of infected mosquitoes resulting from peroral infection using NY10 strains relative to the ancestral WN02. Each NY10 strain infected a greater proportion of mosquitoes than WN02 at every dose tested (ANCOVA, *p* < 0.01, Figure 4). NY10C was found to be the most infectious strain, with an ID_50_ >1 log_10_ pfu/mL lower than that of WN02 and a minimal infectious dose of 4.0 log_10_ pfu/mL. The mean ID_50_ for NY10 strains, 6.05 log_10_ pfu/mL, was 0.80 log_10_ pfu/mL lower than that of WN02.

### Vector competence of WNV NY10 strains in Culex pipiens

**Figure 5.**
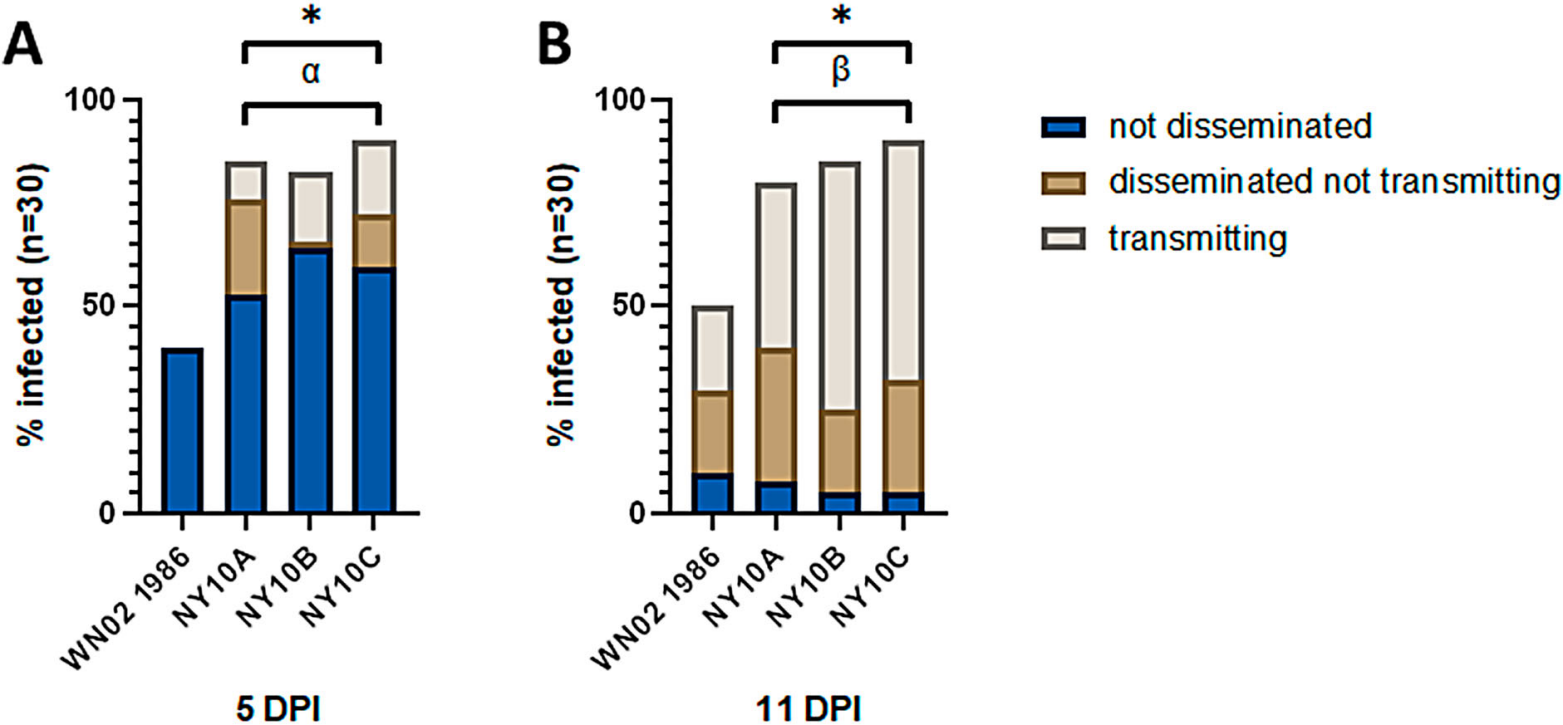
Vector competence of *Culex pipiens* for WN02 and NY10 strains. A) At 5 days post-infection (DPI) *Culex pipiens* infected with NY10 strains showed significantly increased rates of infection and dissemination when compared to WN02. B) At 11 DPI, *Culex pipiens* infected with NY10 strains showed significantly greater rates of infection, dissemination, and transmission than WN02 infected mosquitoes [*p* < 0.05 for infection (*), dissemination (α), and transmission (β) respectively, when compared to WN02 by Fisher’s exact test].

At 5 dpi, the infection rate for the NY10 strains is on average 30% greater than that of WN02, with a significantly greater proportion of mosquitoes with disseminated infections (Fisher’s exact t-test, *p* < 0.0001, Figure 5). Mosquitoes infected with the NY10 strains also showed earlier transmission than WN02, which together with the significant increase in dissemination suggests a shorter EIP. This trend was more pronounced at 11 dpi, when significantly enhanced transmission was measured in *Cx. pipiens* infected with NY10 strains (Fisher’s exact t-test *p* < 0.001, Figure 5). These highly significant differences demonstrate a phenotypic advantage that NY10 strains have over WN02 in terms of competence in *Cx. pipiens* mosquitoes.

### West Nile viremia kinetics in American robins

**Figure 6.**
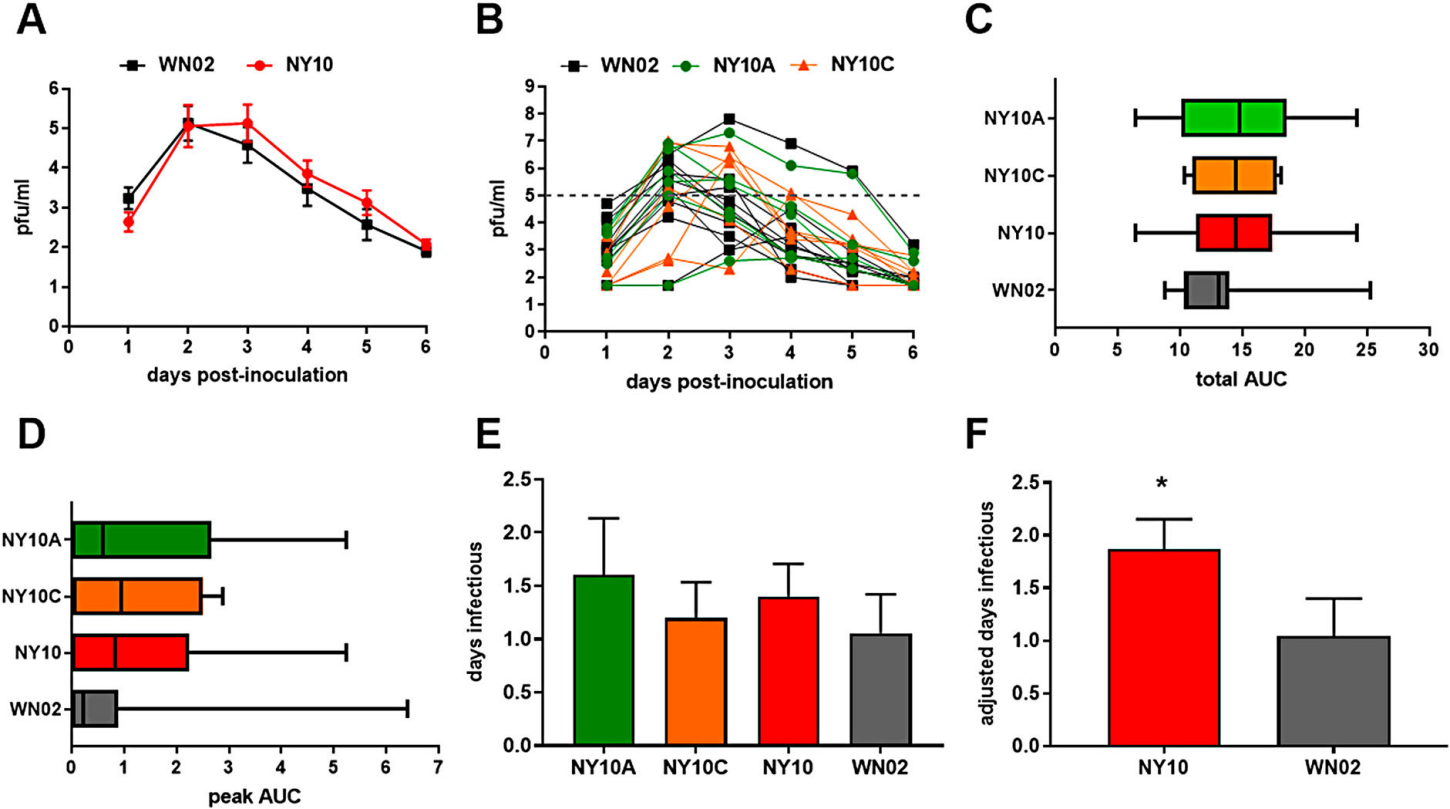
Viremia of American Robins (*Turdus migratorius*) when infected with WN02 and NY10 strains. (**A**) Mean WNV viremia values at each timepoint assayed +/- SEM for WN02 relative to NY10 strains (NY10A/C combined) (**B**) Individual viremia curves for WN02, NY10A and NY10C. The dotted line represents the experimental threshold for infectivity of WN02 to mosquitoes. Mean (vertical line) area under viremia curves (AUC) +/- SEM was calculated for both (**C**) total and (**D**) peak (>5.0 log_10_ pfu/ml). Mean time infectious was calculated using both (**E**) days with viremia >5.0 log_10_ pfu/ml and (**F**) adjusted infectious days based on mosquito studies (Figure 4). Significantly higher mean time infectious was measured for NY10 strains relative to WN02 (*p* = 0.044, Mann-Whitney test).

Overall viremia kinetics were statistically similar for birds inoculated with WNV NY10 strains relative to WN02 (1-way ANOVA, *p* = 0.9971; Figure 6A). However, peak viremia was extended by an average of one day in individuals infected with NY10 strains (Figure 6A). In addition, there was high variability among individuals, yet 5 of 6 birds with the highest peak viremia levels were infected with NY10 strains (Figure 6B). Total viremia (Figure 6C), viremic peak (Figure 6D) and days infectious (Figure 6E) are all higher in NY10 strains compared to WNV 02, though these results were not statistically significant. The individual bird with the highest viremia in the WN02 group represented a significant outlier (paired t-test, *p* = 0.0102). In fact, if this outlier was removed, mean and peak viremia levels would be significantly higher for the birds infected with NY10 strains (paired t-test, *p* < 0.05; Figure 6). While viremia for NY10 and WN02 strains were not independently statistically distinct, when differences in the threshold for mosquito infectivity are considered, days of infectious viremia were significantly higher for NY10 strains (Figure 6F, *p* = 0.044, Mann–Whitney test).

### Increased infectiousness and transmissibility of NY10 strains drives genotype displacement

**Figure 7.**
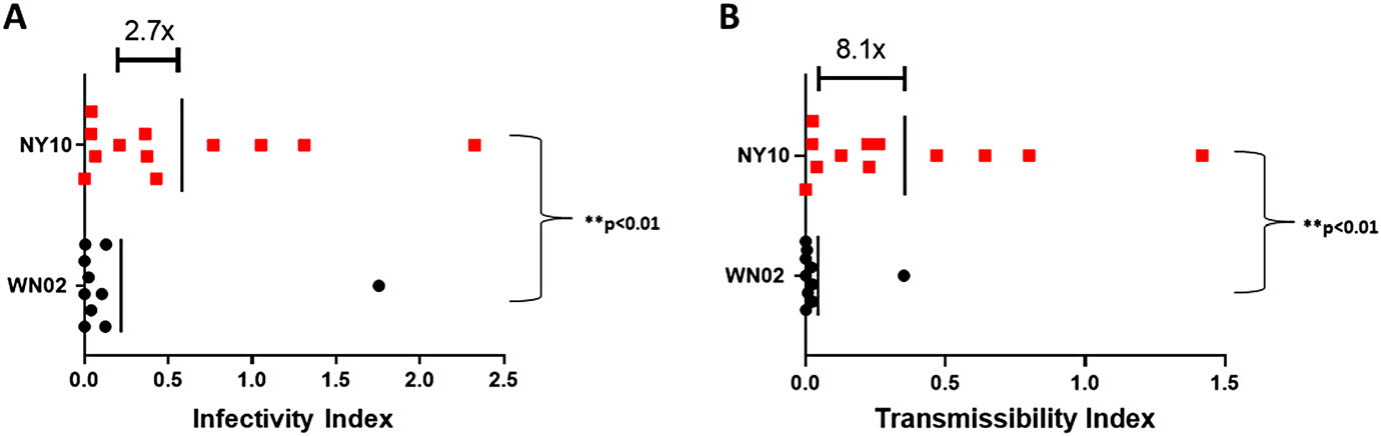
West Nile virus (WNV) infectivity and transmissibility indices. A) Infectivity indices of NY10 strains compared to those of WN02. Points represent individual birds and vertical lines represent means. The average infectivity of NY10 strains was 2.7 times greater than that of WN02 (student’s t-test, *p* < 0.01). B) Transmissibility indices of NY10 strains when compared to WN02. Enhanced transmissibility of NY10 strains further increased the mean difference between genotypes (student’s t-test, *p* < 0.01).

To determine the extent to which phenotypic variation identified for NY10 strains could drive displacement, we quantified infectiousness and transmissibility indices for each experimental infection. Considering distinct viremia kinetics and increased infectivity of mosquitoes (Figures 4 and 6), the average mean infectiousness index for NY10 strains is 2.7 times greater than that of WN02 (Student’s t-test *p* < 0.01, Figure 7). Further, incorporation of transmission data from vector competence results (Figure 5) demonstrates that the mean transmissibility index for NY10 strains is 8.1 times greater than that of WN02 (Student’s t-test *p* < 0.01, Figure 7). Together, these data demonstrate a clear mechanism for displacement of WN02, and increased activity of WNV in NYS since the emergence of NY10.

## Discussion

The markedly increased WNV activity from 2010 to 2018 in NYS coincides with a greater number of human cases, and the rise in prevalence of new, emergent genotypes of WNV, with the most obvious and striking trend being the dominance of the NY10 genotype. The mutations that define this genotype, R1331 K (NS2A R188 K) and I2513M (NS4B I240M), occurred separately in years before 2010, but after 2010 were found largely in tandem, suggesting an adaptive linkage. Based on the distinct clades that NY10 occurs in, it has been selected on at least 2 different backgrounds [25]. Recently, there has been identification of the NY10 genotype on distinctly different, and geographically distant backgrounds (nextstrain.org/wnv/na). While further mechanistic studies are required to fully define the molecular mechanisms resulting in increased vector competence and altered viremia kinetics, the flavivirus genes NS2A and NS4B are known to play important roles in replication, virion assembly and immune evasion in both vertebrate and invertebrate hosts [32–34]. NS2A is a documented suppressor of RNA interference (RNAi) through direct binding and sequestration of the Dicer-2 enzyme in vertebrate hosts and mosquitoes [35]. Increased capacity to act in this regard could enhance viral replication and transmission, particularly in mosquitoes that rely on RNAi as a primary immune response to arboviruses [36]. While the primary phenotype identified here is increased infectivity and transmissibility of the NY10 genotype in *Culex pipiens* mosquitoes, it is possible modest changes in avian viremia could be related to strain-specific variability in the interferon response. There is a documented role for the NS4B as an interferon antagonist, which is known to be strain-dependent [37–40]. Though the position of the NS4B gene substitution has not been previously attributed to this function, it is certainly possible that this could influence the interferon response [38]. The flavivirus protein NS4B, although composed of just 255 amino acids, was previously found to possess the highest number of shared non-synonymous consensus mutations among sequenced WNV isolates, including three positions with evidence of positive selection [25]. The NS4B is known to interact with numerous host and viral proteins with diverse roles in viral replication and host immunity. Concordantly, individual substitutions in this protein are well documented to have the capacity to result in significant changes to host-specific fitness and pathogenesis [37]. Similar to NS2A, NS4B has been identified as an RNAi suppressor [41]. Additionally, NS4B likely contributes to both evasion and/or suppression of the cellular stress response [42,43]. Lastly, because NS4B interacts directly with the replication complex, substitutions could additionally perturb replication kinetics [40,44].

Flavivirus evolution is driven primarily by stochastic change within and between hosts and seasons, and purifying selection in the host and vector, with limited evidence of positive selection or selective sweeps, with the exception of the displacement of the NY99 genotype by the WN02 genotype [19,20,45–47]. The comparison of the WN02 and NY10 genotype strains in *Culex pipiens* clearly demonstrates increased competence for NY10. A similar phenomenon was observed with WN02 strains relative to NY99 strains in *Culex* mosquitoes, which was attributed primarily to the V449A substitution in the envelope gene [16,23]. Surprisingly, additional genotypes possessing enhanced infectivity or transmissibility in *Culex* species mosquitoes have not been observed. The NY10 genotype strains of WNV tested here each differ in their amino acid sequences, with unique substitutions in both structural and non-structural proteins. Included in these differences is the G2377E substitution in the NS4B found in isolate 10C, a mutation previously attributed to the NY01 genotype with evidence of positive selection [25]. Additional distinct substitutions were identified in the prM, E, NS2 and NS5 proteins. While each of these could certainly contribute to altered viral fitness or transmissibility, none were shared among NY10 genotype isolates. Since NY10 strains were all associated with increased competence of *Cx. pipiens* relative to WN02, the presence of the signature NY10 mutations is most likely to be primarily responsible for the fitness advantage. Additionally, while transmission at 5 dpi was detected in individuals in all three groups infected with NY10 strains, none of the WN02 infected mosquitoes transmitted at 5 dpi, indicating a shorter mean EIP and an additional advantage for the propagation of NY10 genotype strains.

The fact that examples of adaptive evolution of WNV and other arboviruses are rare is often attributed to adaptive trade-offs imposed by the disparate selective pressures of vertebrate and invertebrate hosts [48–52]. Here, although the increased fitness in mosquitoes is more pronounced, we additionally demonstrated that more birds infected with NY10 genotype strains had higher mean viremia and longer mean sustained viremic periods. While the protracted viremic period is modest, on a population level it would have a substantial effect on infectiousness, particularly in the context of the significant increase in infectivity to mosquitoes. If such differences exist in other avian hosts, an additional consequence of these mutations could be expansion of host range, where species generally thought to be poorly competent (i.e. doves, non-*Turdus* thrushes, and catbirds) could ultimately play a much larger role in virus amplification [53–55].

Adaptive evolution of WNV occurring in North America two decades after its introduction is somewhat surprising, and perhaps a result of environmental changes and/or shifts in host or vector populations. Previous studies have reported that distinct interactions between viral genotype, mosquito population and temperature influence vector competence [14,56]. Vector populations have relatively fast generation times, restricted geographic ranges, and proposed mechanisms of overwintering in mosquitoes can drive the emergence of new genotypes [57]. It remains unclear whether NY10 strains are additionally more adaptive to *Cx. quinquefaciatus*, *Cx. tarsalis*, or other populations of *Cx. pipiens*. Previous studies did suggest a geographical bias among WNV genotypes, with NY10 more likely to occur in northern NY and the NY07 genotype strains having increased prevalence in downstate NY [25]. The question of differential vector competence between *Culex* species and populations is highly relevant, as these differences in infectivity can greatly shape the nature and magnitude of the spread of WNV, as was the case with *Cx. tarsalis* in the US and other *Culex* species implicated in enhanced WNV transmission in Europe [23,58,59]. Additionally, changes to the environment, particularly increases in temperature associated with climate change, may facilitate broad adaptation, perhaps expanding the host and geographical range of the virus [60]. Importantly, while our previous studies only identified the NY10 genotype in NYS, it is now present throughout the continental US, suggesting that it may have a broad adaptive advantage that could drive similar increases in WNV activity in other regions (nextstrain.org/wnv/na).

These data demonstrate the importance of analyzing spatially or temporally distinct datasets. For traditional phylogenetic studies, the focus is often placed on the size of the dataset, the idea being that more sequence data equate to increased capacity for inference of selection analysis and detection of adaptive change. If selection is variable over space and time a shortcoming of analyzing an expansive dataset is that signals of positive selection or displacement occurring throughout shorter periods or in discrete regions could be diluted with data from other regions when traditional counting methods of selection analysis are used. This can result in relevant evolutionary events being pushed into statistical insignificance.

While NY10 remains the dominant genotype in NYS and has now spread throughout the US, NY07, another previously identified emergent genotype, has also increased in prevalence since its emergence. Further phenotypic characterization and continued WNV surveillance will help elucidate if this and other novel genotypes could facilitate additional regional or national expansions to WNV transmission and disease.

## Supplementary Material

Supplemental Material
