# Supplementary figures and images for: Adaptive evolution of West Nile virus facilitated increased transmissibility and prevalence in New York State
Source: Emerg Microbes Infect. 2022 Mar 31;11(1):988–99. doi: 10.1080/22221751.2022.2056521 (PMC8982463; doi:10.1080/22221751.2022.2056521)

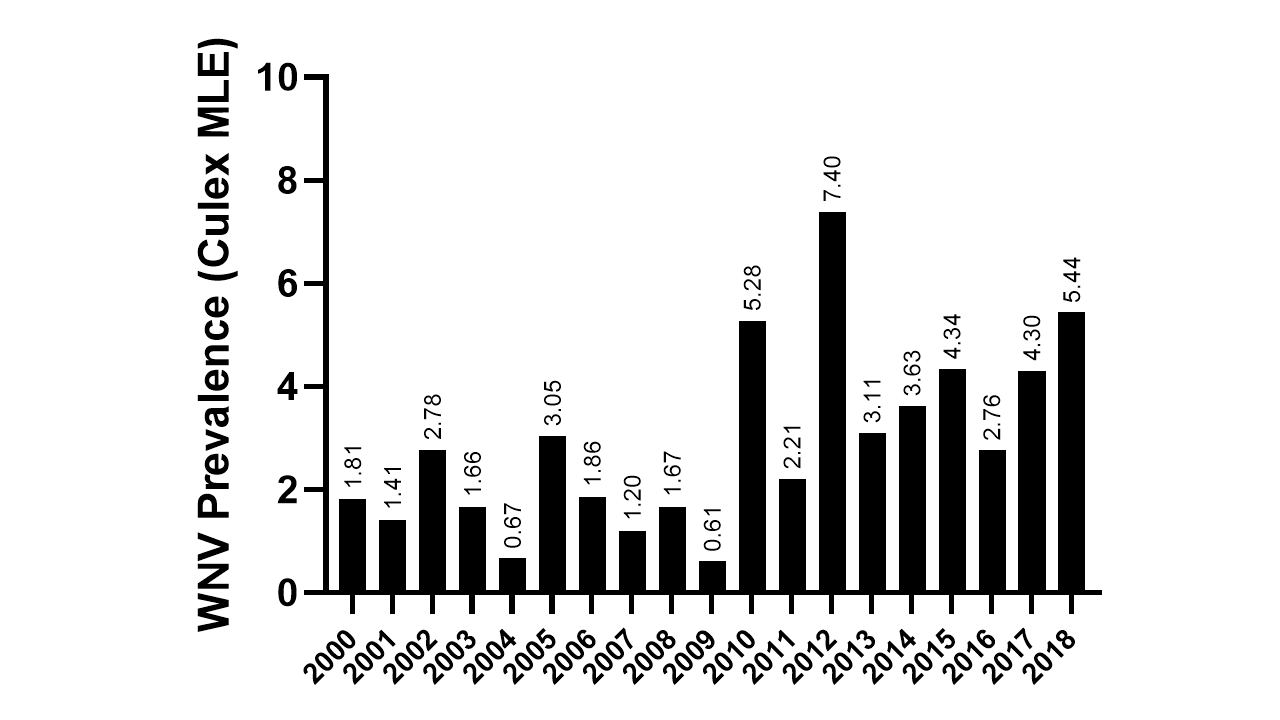

Supplement: Supplemental Material [file TEMI_A_2056521_SM9071.tif]
